# Continuous ambulatory peritoneal dialysis—a guide to imaging appearances and complications

**DOI:** 10.1007/s13244-012-0203-y

**Published:** 2012-12-06

**Authors:** Mark Goldstein, Maria Carrillo, Sangeet Ghai

**Affiliations:** 1 Department of Radiology, John Radcliffe Hospital, Oxford University Hospitals, Headley Way, Oxford, OX3 9DU UK; 2 Joint Department of Medical Imaging, University of Toronto, Toronto General Hospital, 200 Elizabeth St., Toronto, ON M5G 2C4 Canada

**Keywords:** Continuous ambulatory peritoneal dialysis (CAPD), X-ray computed tomography, Ultrasonography, Catheters, Peritonitis

## Abstract

**Objectives:**

The aim of this article is to review and illustrate the typical imaging findings for a patient on continuous ambulatory peritoneal dialysis (CAPD) and its complications, examining the uses and limitations of multimodality imaging.

**Background:**

CAPD is a commonly and increasingly used method of renal replacement therapy in end-stage renal failure (ESRF). From the set-up and insertion of the peritoneal catheter through to the actual treatment, there are pitfalls and complications that may adversely affect the patient and compromise the success of the dialysis. Complications can be either immediate or delayed, and can also be categorised into infectious and non-infectious aetiologies, including catheter failure, dialysate leaks, hernias and encapsulating sclerosing peritonitis.

**Conclusion:**

Early recognition of complications, both clinically and on the different imaging modalities, is essential in the management of CAPD in order to reduce treatment failure and limit patient morbidity and mortality.

**Main messages:**

- *Complications of peritoneal dialysis cause patient morbidity and treatment failure.*
- *Early recognition of complications from normal appearances is essential to limit dialysis failure.*
- *Multimodality imaging plays an important role in the diagnosis of these complications.*

## Introduction

Renal replacement therapy is a rapidly evolving specialty. For patients with end-stage renal failure (ESRF) there are a limited number of options. They include peritoneal dialysis (PD), haemodialysis (HD) and, with increasing frequency, renal transplant, where available. By the end of 2005, the number of patients undergoing treatment for ESRD was estimated to have reached 1.9 million worldwide, of which 1.455 million were being treated by dialysis and the remaining 445,000 were living with a functional renal graft. Of the global dialysis population in 2005, 89 % underwent HD treatment and only 11 % were treated by PD. This equates to a global total approaching 160,000 at this time [1].

With the aim to optimise patient outcomes and quality of life, renal transplant if successful is in most cases the desired option. This is, however, not widely available due to limitations in the availability of organ donors and also appropriately skilled and trained surgeons. In these cases the patient has the option of HD and CAPD with the pros and cons different for both with CAPD utilised in approximately one-third of new ESRF cases in the US and Europe [2].

**Fig. 1:**
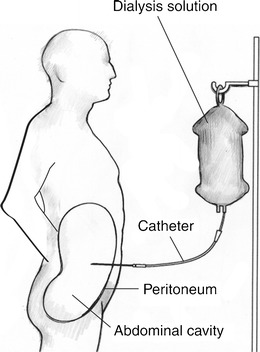
Illustration of the normal CAPD setup with catheter position normally ending with the tip in the pelvis. (Image with permission from National Institute of Diabetes and Digestive and Kidney Diseases, National Institutes of Health)

The technique of continuous ambulatory peritoneal dialysis (CAPD) was first described in the 1950s [3] and became more widely used in the 1970s [4]. It is typically performed intermittently up to four times daily with injection of the dialysate into the peritoneal cavity via a trans-abdominal catheter entering through the anterior abdominal wall, piercing the parietal peritoneum and with its tip sited in the pelvis. The peritoneal membrane is then utilised for the exchange of electrolytes, glucose, urea, albumin and other small molecules from the blood (Fig. 1). Typically 2–2.5 l dialysate are injected into the peritoneal cavity distributing mainly throughout the greater sac and also into the lesser sac via the Foramen of Winslow with resultant increase in intra-abdominal pressure which can contribute to complications of the treatment. It is important that the dialysate distributes throughout the peritoneal cavity and utilises the available peritoneal membrane surface area. With the pelvis holding the greatest volume of dialysate, the success of CAPD can be compromised by any complications that restrict this distribution such as adhesions [5].

The main advantages of CAPD are speed and ease of use, relative inexpense, absence of need for a highly skilled operator and lack of need for anticoagulation. It is preferred where vascular access is challenging or in patients with cardiovascular disease as it induces less cardiovascular stress [5-7].

Disadvantages include many potential complications, some of which occur more frequently with CAPD than other renal replacement techniques. These may be medical, such as electrolyte/acid–base imbalance or infection, surgical or mechanical catheter related. The most common cause for failure of treatment is infection due to the in-dwelling catheter; however, there are many causes for treatment failure such as dialysate leaks, hernias, intestinal obstruction and most seriously encapsulating sclerosing peritonitis (ESP) [5].

CAPD was at one stage the treatment of choice for both acute (ARF) and chronic renal failure (CRF) before advances in HD and continuous renal replacement therapies (CRRT) demonstrated improved outcomes, making it no longer the treatment of choice in ARF patients. There are several relative contraindications to consider with CAPD, including recent abdominal or cardiothoracic surgery, diaphragmatic pleuro-peritoneal connections, peritonitis, severe respiratory failure and abdominal wall cellulitis [4]. Alternative uses for PD other than renal replacement therapy have been seen for the treatment of acute pancreatitis [8] and hyperthermia/hypothermia [9-11], which have had limited success and are not routinely in clinical use.

## Imaging techniques and normal appearances

Plain film radiography is cheap and readily available but of limited value. It can be used to identify catheter position, which should normally follow a path once inside the abdominal cavity caudally into the pelvis, and some complications such as ESP when advanced, perforation and hydrothorax if a pleura-peritoneal communication exists. It is, however, less sensitive and specific than computed tomography (CT) for diagnosing dialysate leaks and other complications, but can still be used as a first line in the investigation of complications [12].

Ultrasound with its lack of ionising radiation is a highly useful test that can identify many complications such as abdominal wall or intra-abdominal collections, peritoneal thickening, peritoneal calcification or thickened and dilated small bowel loops but has limitations due to operator dependence and lack of sensitivity to rule out pathologies such as focal peritoneal thickening seen in early SP compared with CT [12].

**Fig. 2:**
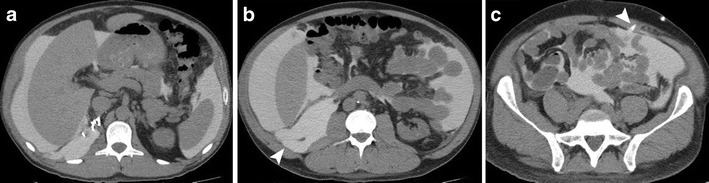
Axial CT peritoneography in a patient with prior right nephrectomy demonstrating peritoneal distribution of dialysate mixed with iodinated contrast in the upper abdomen (**a**) communicating into the lesser sac anterior to the pancreas. At a more inferior level in the mid-abdomen (**b**) with dialysate seen within the right retroperitoneum at the nephrectomy bed (*arrowhead*) as a consequence of prior surgery, and in the pelvis (**c**) surrounding large and small bowel loops and within the mesentery with a Tenckhoff dialysis catheter seen entering the peritoneal cavity through the left rectus muscle (*arrowhead*)

CT can be performed both with intravenous (IV) and oral contrast, or more usefully to evaluate for complications of CAPD with additional injection of an iodinated contrast medium into the peritoneal cavity via the in-dwelling catheter, which is a technique known as CT peritoneography (Fig. 2) the indications for which are listed in Table 1 [13]. Dialysate is drained from the peritoneal cavity then 1 ml/kg of non-ionic contrast material such as iodine 300 mg/ml is mixed with 30 ml/kg of dialysate fluid and injected into the peritoneal cavity via the dialysis catheter. The patient is then asked to mobilise and walk around before the scan is performed approximately 30 min later after the mixture has had time to distribute around the peritoneal cavity in the supine position though decubitus and prone positioning may also be utilised if supine images do not demonstrate any abnormality [5].

**Table 1:** Indications for CT peritoneography

Ultrafiltration failure
Difficulty with fluid exchange
Recurrent peritonitis leading to adhesions and loculated peritoneal fluid collection or abscess
Abdominal wall swelling or soft tissue oedema suggesting dialysate leak
Hernias—umbilical, abdominal wall, inguinal

Dialysate distribution in the supine position within the peritoneal cavity has been estimated to be approximately 30–55 % within the pelvis surrounding mesentery and bowel, 10–20 % in the upper abdomen in perihepatic and perisplenic location and only 1–3 % within the lesser sac [13]. CT peritoneography has been shown to be superior to standard CT for the diagnosis of complications such as dialysate leaks, the path and site of which can be better delineated, and intra-abdominal abscesses, which appear as unopacified fluid collections within the peritoneal cavity [6, 13].

Use of IV and oral contrast is not routinely required and can be used selectively for indications such as suspected intestinal obstruction, or inflammatory aetiologies such as pancreatitis or bowel ischaemia. However, IV contrast has the disadvantage of potential nephrotoxicity in patients with residual renal function and its use therefore needs to be evaluated in the context of risk-benefit to the patient by the attending physician. Also, altered enhancement of structures such as small or large bowel surrounded by the opacified dialysate may be obscured if CT peritoneography is performed.

Magnetic resonance imaging (MRI) can be of value with its greater soft tissue contrast, lack of ionising radiation exposure and multiplanar capability. It can be performed with or without a mixture of a gadolinium-based contrast agent into the dialysate and then similar to CT peritoneography, allowing a 30-min delay and the patient to mobilise to achieve adequate distribution of the fluid. T1-weighted imaging can then be utilised with no IV or oral contrast required. Alternatively, T2-weighted imaging can be utilised to delineate fluid collections without the use of gadolinium. However, in addition to cost and lack of availability, the spatial resolution is inferior to CT and given the uncertain potential to induce nephrogenic systemic fibrosis from gadolinium exposure within the peritoneal cavity this technique is not routinely recommended [2].

Scintigraphy with intra-peritoneal injection of a radioisotope has been utilised in the imaging of hernial complications related to CAPD prior to the widespread use of CT peritoneography for this purpose [14] or to detect the presence of a pleuro-peritoneal fistula [15], but is not routinely used in clinical practice.

## Complications

### Infections

Bacterial infections causing peritonitis are the commonest occurring complication, with a reported frequency of one episode every 20–30 months per patient [5] and are the commonest cause of catheter replacement [16]. The likeliest route of infection is via the dialysis catheter but can occur secondary to other intra-abdominal infective or inflammatory pathologies such as cholecystitis or diverticulitis [7].

**Fig. 3:**
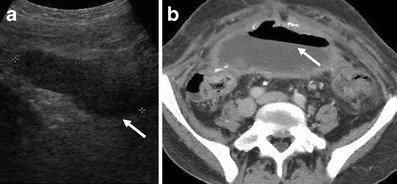
Anterior pelvic abscess seen on ultrasound (**a**) as complex fluid collection (*arrow*) containing debris and axial CT (**b**) showing an air-fluid level (*arrow*) in a patient on long-standing CAPD with peritoneal calcifications from encapsulating sclerosing peritonitis

**Fig. 4:**
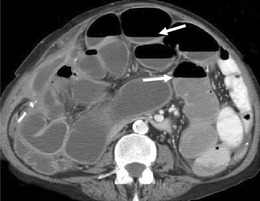
Multiple dilated small bowel loops seen on axial CT with IV and oral contrast with several air-fluid levels (*arrows*) and positive oral contrast seen only within proximal small bowel loops left side of the abdomen in keeping with intestinal obstruction in a patient who developed adhesions as a complication of peritoneal dialysis

Imaging of peritonitis is limited though the complications such as collections with wall enhancement, mesenteric inflammatory change, thickening of the small bowel wall or adhesions with their associated sequelae such as obstruction or ischaemia may be seen (Figs. 3, 4). Adhesions from prior episodes of peritonitis are the most frequent cause of maldistribution of dialysate, causing both dialysis failure and other complications including intestinal obstruction sometimes requiring surgical intervention [5]. The presence of infection within a collection cannot always be determined on imaging appearances alone and aspiration may be required for diagnosis. Residual dialysate fluid when present has the potential to obscure some of the inflammatory changes in the mesentery or create difficulty in interpretation, as it may be the cause of thickening of the intestinal or gallbladder wall.

**Fig. 5:**
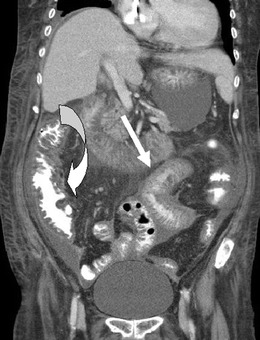
TB peritonitis in a CAPD patient presenting with non-specific abdominal symptoms and general malaise confirmed by microbiological evaluation of the dialysate seen on coronal CT with IV contrast demonstrating stranding of the mesentery, small (*straight arrow*) and large (*curved arrow*) bowel wall thickening features of which are non-specific and can also be seen with other causes of peritonitis

TB infections are frequently seen in CAPD patients due to reduced cellular immunity. The imaging features overlap to a large extent with other causes of peritonitis, and the diagnosis requires microbiological evaluation of dialysate fluid, occasionally peritoneal biopsy as well as clinical history (Fig 5) [17, 18].

**Fig. 6:**
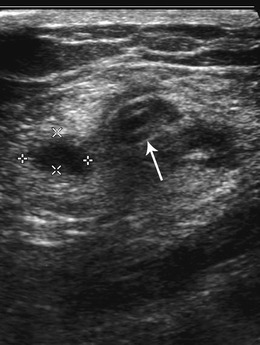
Tunnel infection seen in a patient with abdominal wall erythema and pain on ultrasound with a small hypoechoic collection adjacent to the catheter which is seen as parallel hyperechoic lines (*arrow*) within the anterior abdominal wall

Infections can also involve the extra-peritoneal structures of the abdominal wall, particularly at the catheter exit site. Tunnel infection within the anterior abdominal wall soft tissues may cause pain and associated visible erythema at the skin surface, and may be accompanied by an adjacent subcutaneous collection or abscess, which can be picked up on ultrasound (Fig 6) [18].

### Catheter failure

**Fig. 7:**
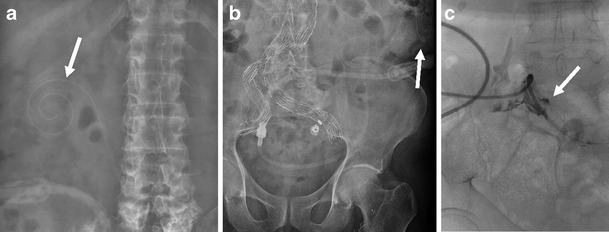
Abdominal radiographs demonstrating catheter tip migration superiorly from the pelvis to the right (**a**) and left (**b**) upper abdomen (*arrows*) in different patients and entrapment of the tip in another patient (**c**) with prevention of water soluble contrast from spilling freely into the peritoneal cavity on manual injection under fluoroscopy (*arrow*)

Catheter failure can occur due to malposition, kinking or entrapment. Migration of the catheter from the pelvis displaces the catheter tip away from the greatest volume of the dialysate (Fig. 7). Manual manipulation of the catheter back into position can be attempted and if unsuccessful then this can be done under fluoroscopic guidance [18]. Entrapment can occur secondary to peritonitis with adhesions or to adherent omentum at the tip (Fig. 7). Rarely, insertion of the catheter may be complicated by a perforated viscera.

### Dialysate leaks

Dialysate leakages as a complication occur in >5 % of patients and are often not of clinical significance [19]. They can be classified as early at ≤30 days, where the likely aetiology is catheter related, or late at >30 days, likely due to a mechanical or surgical tear in the peritoneal membrane. With increased intra-abdominal pressure from the infusion of dialysate there is increased likelihood of a leak from the peritoneal cavity as well as respiratory compromise from splinting of the diaphragm. Other mechanisms that raise intra-abdominal pressure, such as coughing, straining or obesity, can also predispose to leaks [19, 20].

**Fig. 8:**
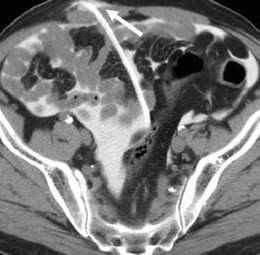
CT peritoneography axial image demonstrating a dialysate leakage at the catheter insertion site within the anterior abdominal wall (*arrow*)

**Fig. 9:**
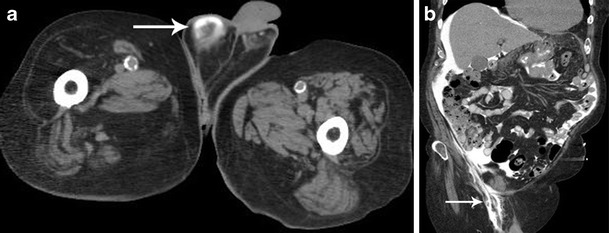
CT peritoneography axial (**a**) and oblique coronal (**b**) images demonstrate dialysate leakage via a patent right processus vaginalis into the right scrotal sac (*arrows*)

**Fig. 10:**
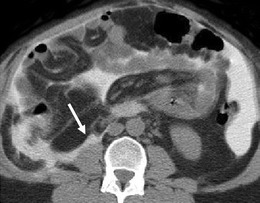
CT peritoneography axial image demonstrates a rare case of dialysate leakage into the right retroperitoneum with dialysate seen anterior to the right psoas muscle (*arrow*)

The entry point of the catheter creates an obvious weak point, as does any previous surgical defect in the peritoneal wall (Fig. 8). Other potential sites of leakage are within the abdominal wall at prior surgical defects, via pleuro-peritoneal connections into the thoracic cavity presenting with pleural effusions or into the scrotum via a patent processus vaginalis (Fig. 9) [18]. Rarely, a retroperitoneal leak may occur which is unlikely to be detected clinically, however, is of significance as it can be the cause of acute ultra-filtration failure, which may necessitate stopping CAPD treatment for a period and in some cases requires surgical intervention (Fig. 10) [21].

### Hernias

**Fig. 11:**
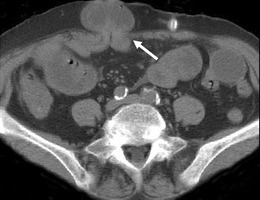
Axial unenhanced CT of a CAPD patient with a ventral hernia (*arrow*) at a site of previous catheter insertion containing an incarcerated loop of small bowel causing a mechanical obstruction that required surgical intervention

Hernias are a frequent occurrence as a consequence of the raised intra-abdominal pressure and peritoneal defect created by the catheter insertion and are seen in up to 25 % of CAPD patients, with the commonest at the umbilicus, adjacent to the catheter and at the inguinal canal [7]. They may be complicated by a dialysate leak or, as with all hernias, can cause incarceration and strangulation of bowel loops, which may need emergency surgical intervention (Fig. 11).

### Encapsulating sclerosing peritonitis

Encapsulating sclerosing peritonitis (ESP) also referred to as sclerosing peritonitis is an inflammatory process leading to the deposition of a thick fibrous membrane on the peritoneum, occurring in approximately 1 % of patients on CAPD overall, with prevalence increasing with length of CAPD treatment, up to nearly 20 % at 8 years [22]. The aetiology of ESP is unclear, but risk factors for its development include increasing length of CAPD—typically for several years, type of dialysate and peritonitis episodes—both bacterial and chemical [23, 24]. Its early diagnosis is important, as cessation of CAPD can prevent progression and further complications of this condition.

Early changes include peritoneal thickening and calcification with non-specific clinical presentations, such as colicky abdominal pain or ultrafiltration failure. It is extremely important to detect early visceral and parietal involvement, as this would indicate the potential for the development of bowel complications. Though overall less sensitive than CT, ultrasound performed with the presence of dialysate fluid has been shown to be sensitive for the diagnosis of ESP, with dilated peristalsing small bowel loops seen earliest and other findings of peritoneal thickening both visceral and parietal, and fibrinous strands also possible to diagnose [25].

**Fig. 12:**
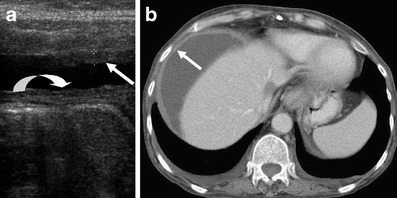
Peritoneal thickening seen on ultrasound (**a**) of the abdomen demonstrates parietal (*straight arrow*) and visceral (*curved arrow*) peritoneal thickening separated by a small amount of free fluid. Axial IV contrast enhanced CT (**b**) portal venous phase in the same patient demonstrates parietal peritoneal thickening (*arrow*) in the upper abdomen

Peritoneal thickness has been found to have a positive correlation with length of treatment of CAPD and can be easily and non-invasively assessed by abdominal ultrasound (Fig. 12). Though a feature of ESP, it is non-specific and can also be seen to increase in thickness with age, body height and weight [26]. An exact thickness cut-off point beyond which CAPD should be discontinued is uncertain and if ESP is suspected due to the findings of peritoneal thickening, a biopsy can be considered, though this has potential complications of an invasive procedure.

**Fig. 13:**
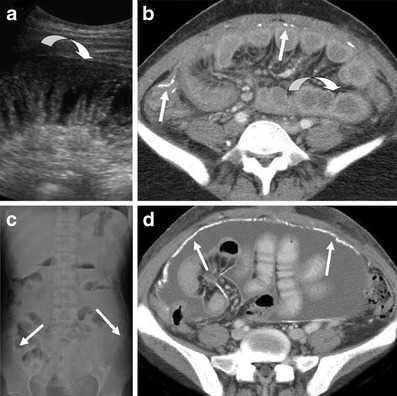
Early ESP changes demonstrated on (**a**) ultrasound with thickening of the wall of a dilated small bowel loop (*curved arrow*) and on axial contrast enhanced CT (**b**) with mild peritoneal calcification (*straight arrows*) and also thickening of the dilated small bowel (*curved arrow*). Two years later in the same patient advanced ESP changes are seen on plain abdominal radiograph (**c**) demonstrating widespread peritoneal calcification (*arrows*). Axial CT image (**d**) demonstrates increased peritoneal calcification and early ‘cocooning’ of the small bowel loops centrally by a fibrous peritoneal sheath illustrating the progressive nature of ESP

Peritoneal calcification both parietal and visceral may or may not occur and typically becomes more extensive over time and can sometimes be seen on abdominal radiographs and ultrasound, but CT is more sensitive, particularly in the early stages (Fig. 13) [25]. Care must be taken not to misdiagnose ESP if the cause of peritoneal calcification is from other aetiologies such as TB infection or rarer conditions such as peritoneal mesothelioma. Later in ESP there is encapsulation of the outer wall of the small bowel by the fibrotic peritoneum with thickening and contraction of the mesentery, which can trap the small bowel centrally, leading to a ‘cocooning’ of the small bowel with acute or sub-acute obstruction (Fig. 13d) [23].

Rarely, ESP presents at a time after the cessation of CAPD. When advanced, treatment is difficult and therefore early diagnosis can lead to a switch in renal replacement therapy and possible prevention of progression, which can ultimately lead to significant morbidity and mortality. The condition is not reversible, though immunosuppression has been reported to have some benefit, and is only amenable to surgical therapy to treat for acute complications such as small bowel obstruction [24].

### Subcapsular hepatic steatosis

**Fig. 14:**
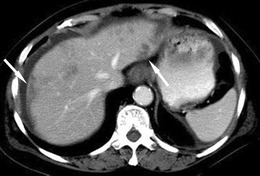
Subcapsular hepatic steatosis seen on axial CT post-IV contrast portal venous phase with subcapsular hypodense areas (*arrows*) throughout the liver in a patient on CAPD. (Image courtesy of Korosh Khalili, Toronto General Hospital, Toronto, Canada)

Subcapsular hepatic steatosis is a rare form of reversible accumulation of fat within the liver, first described in 1989 by Wanless et al. [27], with a reported frequency of 18 % in CAPD patients [28]. It is caused by the exposure of subcapsular hepatocytes to insulin within the dialysate injected into the peritoneal cavity. It can be identified on ultrasound as subcapsular echogenic nodules or rinds without mass effect, as subcapsular hypodense areas on CT and areas that demonstrate signal drop on out of phase T1-weighted MRI images (Fig. 14). Cessation of CAPD has been demonstrated to lead to reversal of these findings [28]. It does not typically cause liver dysfunction and is not of clinical significance however it is important to recognise to prevent further unnecessary imaging.

## Conclusion

CAPD plays an integral role in renal replacement therapy with its many advantages to patients; however, there are significant adverse risks associated with it, leading to serious risk of morbidity and mortality. Early recognition of complications, both clinically and on the different imaging modalities, is essential in the management of CAPD in order to reduce treatment failure and limit patient morbidity and mortality.
